# Novel trypanosomatid species detected in Mongolian pikas (*Ochotona pallasi*) and their fleas in northwestern China

**DOI:** 10.1186/s13071-024-06216-6

**Published:** 2024-03-22

**Authors:** Shiyi Wang, Suwen Wang, Xiaoshuang Han, Sándor Hornok, Huiqian Wang, Nan Wang, Gang Liu, Meihua Yang, Yuanzhi Wang

**Affiliations:** 1 Key Laboratory for Prevention and Control of Emerging Infectious Diseases and Public Health Security, the XPCC, School of Medicine, Shihezi University, Shihezi, Xinjiang, Uygur Autonomous Region China; 2 Department of Parasitology and Zoology, University of Veterinary Medicine, Budapest, Hungary; 3 HUN-REN-UVMB Climate Change, New Blood-Sucking Parasites and Vector-Borne Pathogens Research Group, Budapest, Hungary; 4 College of Agriculture, Shihezi University, Shihezi, Xinjiang Uygur Autonomous Region Republic of China

**Keywords:** *Trypanosoma*, *Blechomonas*, *Frontopsylla*, Central Asia

## Abstract

**Background:**

In the family Trypanosomatidae, the genus *Trypanosoma* contains protozoan parasites that infect a diverse range of hosts, including humans, domestic animals, and wildlife. Wild rodents, as natural reservoir hosts of various pathogens, play an important role in the evolution and emergence of Trypanosomatidae. To date, no reports are available on the trypanosomatid infection of pikas (Lagomorpha: Ochotonidae).

**Methods:**

In this study, Mongolian pikas and their fleas were sampled at the China–Mongolia border, northwestern China. The samples were analyzed with polymerase chain reaction (PCR) and sequencing for the presence of Trypanosomatidae on the basis of both the 18S ribosomal RNA (*18S rRNA*) gene and the glyceraldehyde-3-phosphate dehydrogenase (*gGAPDH*) gene. The morphology of trypomastigotes was also observed in peripheral blood smears by microscopy.

**Results:**

Molecular and phylogenetic analyses revealed a new genotype of the *Trypanosoma lewisi* clade that was found both in pika blood and flea samples. This genotype, which probably represents a new species, was provisionally designated as “*Trypanosoma* sp. pika”. In addition, a novel genotype belonging to the genus *Blechomonas* of Trypanosomatidae was detected in fleas. On the basis of its molecular and phylogenetic properties, this genotype was named *Blechomonas luni*-like, because it was shown to be the closest related to *B. luni* compared with other flea-associated trypanosomatids.

**Conclusions:**

To the best of our knowledge, this is the first study to report any trypanosomatid species in Mongolian pikas and their fleas. Further studies are needed to investigate the epidemiology of these protozoan parasites, as well as to evaluate their pathogenicity for humans or domestic animals.

**Graphical Abstract:**

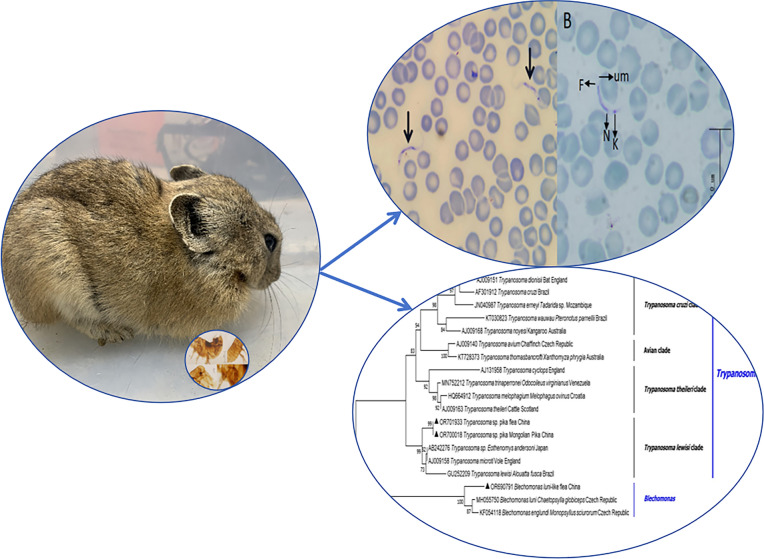

**Supplementary Information:**

The online version contains supplementary material available at 10.1186/s13071-024-06216-6.

## Background

Currently, the family Trypanosomatidae (Euglenozoa: Kinetoplastida) includes 24 genera, as exemplified by *Trypanosoma*, *Leishmania*, *Crithidia*, *Leptomonas*, and *Blechomonas* [1]. *Trypanosoma* species are parasites found in a broad range of domestic and wild vertebrate hosts (such as horses, deer, elephants, camelids, equines, buffaloes) and are transmitted by blood-sucking arthropod (e.g., fly, tick, and flea) or leech vectors [2]. Human pathogenic trypanosomes include *Trypanosoma brucei gambiense* and *T. brucei rhodesiense* causing sleeping sickness in Africa and *T. cruzi* as the etiological agent of Chagas disease in South America [2]. These, as well as other *Trypanosoma* species (e.g., *T. evansi*, causing surra) are also important in veterinary medicine. Taxonomically, the genus *Trypanosoma* is divided into six groups: the *T. cruzi* clade, the *T. brucei* clade, the *T. pestanai* clade, the *T. irwini* clade, the *T. theileri* clade, and the *T. lewisi* clade [1, 3].

Pikas (Lagomorpha: Ochotonidae) are small, rabbit-like mammals with Holarctic distribution. This family of lagomorphs is composed of a single genus, *Ochotona*, including 30 species [4]. The majority of pika species are found in West and Central Asian countries. The Mongolian pika (*Ochotona pallasi*) is only indigenous to a small area at the junction of three countries: China, Mongolia, and Russia [5]. Living in the alpine meadow ecosystem, it is closely associated with a broad range of wildlife, as well as domestic animals and humans [6]. However, epidemiological research on Mongolian pikas was almost exclusively conducted in the context of *Yersinia pestis*, and information on the occurrence and prevalence of Trypanosomatidae species in these lagomorphs is still scarce.

The aim of the present study was to uncover Trypanosomatidae species and their potential vectors at the China–Mongolia border in northwestern China.

## Methods

### Sample collection and identification

Between 2021 and 2023, a total of 83 Mongolian pikas were collected at 15 sampling sites in Beitashan Mountain (coordinates: 45.37° N, 90.53° E, elevation: 1653.7 MASL) at the border region near Mongolia in northwestern China (sampling sites are shown in Additional file 1). These pikas were captured by Sherman traps, which were placed at the entrances of occupied burrows. Each survey site had 150 traps that were checked twice daily [7]. Fleas were collected from individual Mongolian pikas by brushing their fur. Blood smears were prepared from the peripheral circulation, stained with Giemsa. In addition, the heart, liver, spleen, lung, and kidneys were removed. Simultaneously, fleas were collected from the body surface of each pika. All fleas were morphologically and molecularly identified according to our previous work [8]. The fleas were later allocated into pools ranging from 2 to 6 specimens on the basis of species. In this way, a total of 20 flea pools were analyzed.

### Detection, sequencing, and phylogenetic analysis

DNA extractions from the blood samples and fleas were carried out using the TIANamp Genomic DNA Kit (TIANGEN, Beijing, China). PCR targeting 850-bp-long part of 18S ribosomal RNA gene (*18S rRNA*) of Trypanosomatidae was also performed. The primer sequences were as follows: rrf-OF: 5ʹ-CACCCGCGGTAATTCCAGC-3ʹ, and rrf-OR: 5ʹ-CTGAGACTGTAACCTCAA-3ʹ [9]. The PCR cycling conditions for Trypanosomatidae detection consisted of an initial 5-min denaturation at 94 ℃, followed by 35 cycles at 94 ℃ for 40 s, 60 ℃ for 40 s, and 72 ℃ for 40 s, with a final extension at 72 ℃ for 10 min. To confirm positivity of PCR, it was attempted to amplify an additional genetic marker, an approximately 820-bp-long fragment of the *gGAPDH* gene, encoding glyceraldehyde-3-phosphate dehydrogenase of trypanosomes. The outer reaction of this nested PCR was conducted with primers gGAPDH-F1: 5ʹ- CTYMTCGGNAMKGAGATYGAYG-3ʹ and *gGAPDH*-R1: 5ʹ- GRTKSGARTADCCCCACTCG-3ʹ. The second round PCR was performed with inner primers *gGAPDH*-F and *gGAPDH*-R2: 5ʹ-GTTYTGCAGSGTCGCCTTGG-3ʹ [10]. The PCR conditions were the same as in the *18S rRNA* PCR, except that the annealing temperature was 50 ℃.

Fleas were identified molecularly by targeting an approximately 1000-bp-long part of the 18S ribosomal RNA gene (*18S rRNA*). Primer sequences were as follows: *18S rRNA*-F (5ʹ-CCTGAGAAACGGCTACCACATC-3ʹ) and *18S rRNA*-R (5ʹ-GCATCACAGACCTGTTATTGC-3ʹ) [11]. The conditions of the flea-specific PCR were the same as in the *18S rRNA* PCR of Trypanosomatidae, except that the annealing temperature was 60 ℃. The PCRs were run in a Mastercycler X50s equipment (Eppendorf, Germany).

All PCRs were performed including a sequence-verified positive control and negative control (double-distilled water). PCR products were electrophoretically separated in a 1.5% (*w*/*v*) agarose gel stained with GoldView II, purified using the TIANgel Midi Purification Kit (Tiangen, Beijing, China) and sequenced. Sequences were compared with GenBank data using the BLAST program (http://www.ncbi.nlm.nih.gov/BLAST/). New sequences were submitted to GenBank (accession number for flea *18S rRNA* gene: OR701822; for trypanosome *18S rRNA* gene: OR690791, OR700018, and OR701933; for trypanosome *gGAPDH* gene: PP199391-PP199393). Phylogenetic trees were constructed using the neighbor-joining method in MEGA 7.0 software.

## Results

### Peripheral blood smear analysis

**Figure 1:**
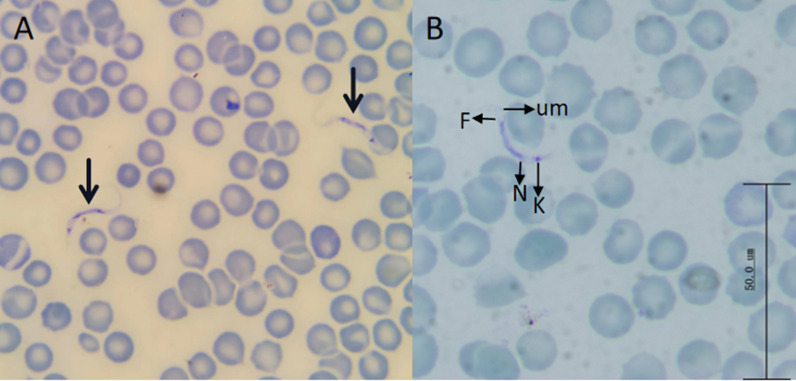
Light microscopy of Giemsa-stained trypomastigotes from Mongolian pika (**A**, **B**). Abbreviations: *N* - nucleus; *K* - kinetoplast; *Um* - undulating membrane; *F* - flagellum)

In the blood smears, fusiform trypomastigotes were observed that possessed a free-flagellar end and undulating membrane. The cell body measured 15–30 μm in length and had a width of 2–5 μm. The nucleus was located in the middle of the cell body, and the kinetoplast close to the rear end (Fig. 1).

### Flea identification

A total of 112 fleas (18 males and 94 females) were identified as *Frontopsylla elatoides elatoides* on the basis of their morphology and 99.6% sequence identity (990/994 bp) to the *18S rRNA* gene of this flea species collected from long-tailed ground squirrel (*Spermophilus undulatus*) in China (KY593303).

### Molecular and phylogenetic analyses

**Figure 2:**
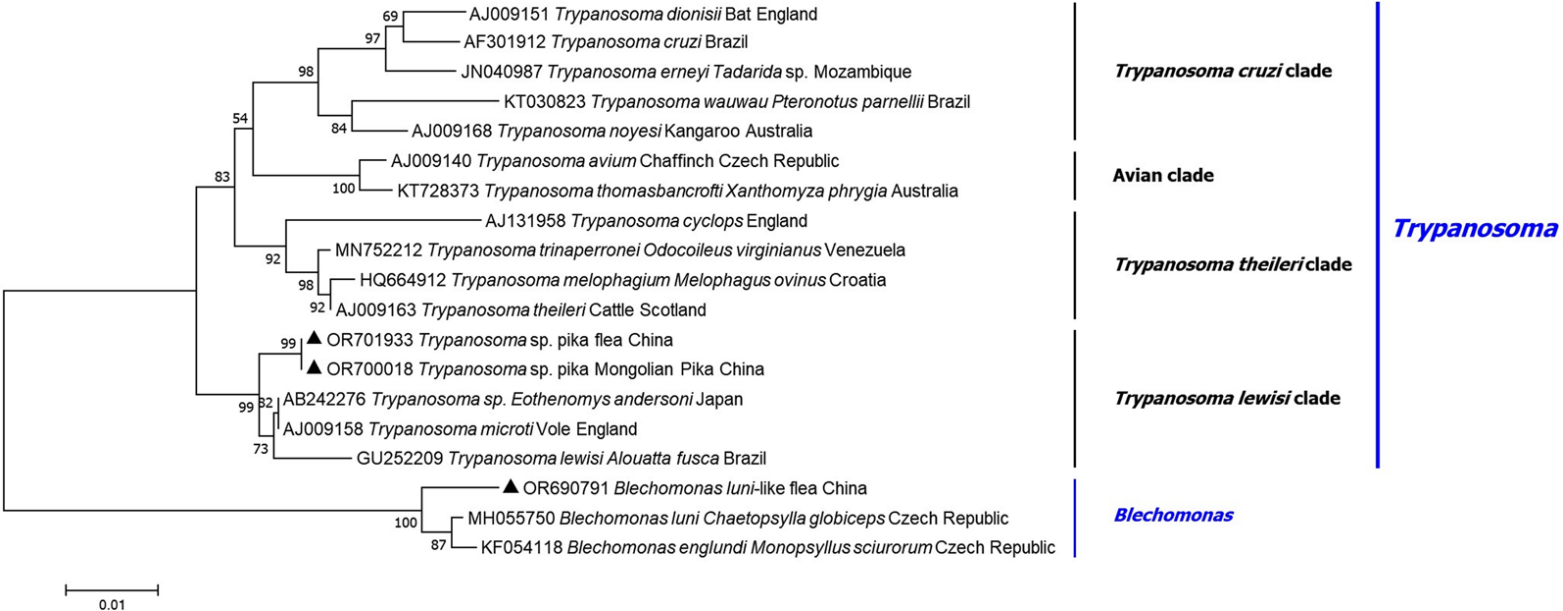
Phylogenetic tree of Trypanosomatidae species from Mongolian pikas and their fleas based on the *18S rRNA* gene. The new sequences provided in the present study are indicated by a black triangle (followed by the accession number)

The DNA of Trypanosomatidae was successfully amplified from the blood of each Mongolian pika. The corresponding *18S rRNA* sequence was closest related to a *Trypanosoma* sp*.* from Anderson’s red-backed vole (*Eothenomys andersoni*) from Japan (AB242276), and to *T. microti* from field vole (*Microtus agrestis*) from England (AJ009158), showing 99.5% (830/834 bp) identity. This new genotype was provisionally named as *Trypanosoma* sp. pika. Phylogenetically, it clustered in the clade of *T. lewisi* (Fig. 2), but separately from other species, with high (99%) support. The *gGAPDH* sequence of this new genotype was closest related to *T. lewisi* from *Rattus omanicus* sampled in Indonesia (LC369597) and to *T. microti* from field vole (*Microtus agrestis*) from England (AJ620273), showing 97.3–98.6% identity (Additional file 2).

Out of the five PCR-positive flea pools, one was confirmed to contain the DNA of *Trypanosoma* sp. pika, showing 100% sequence identity to the genotype detected in the blood of pikas. The trypanosomatid species in the remaining four PCR-positive flea pools was identified as *Blechomonas luni*-like. This genotype was closest related to *B. luni* from the flea *Chaetopsylla globiceps* in Czechia (MH055750), sharing 98.3% (953/970 bp) identity. The *gGAPDH* sequence of this genotype was closest related to *B. luni* from *Chaetopsylla* sp. (KF054103) and to *B. englundi* from *Monopsyllus sciurorum* (KF054101) sampled in the Czechia, showing 87.5–88.7% identity. The phylogenetic clustering of the latter received high (100%) support and was confirmed among other flea-associated *Blechomonas* spp. (Fig. 2).

## Discussion

Most studies mainly focus on domestic and companion animals as the reservoirs of Trypanosomatide [12], but wildlife is also being increasingly considered as an important source of emerging and/or reemerging trypanosomes via vector-borne transmission [13, 14]. However, among wild-living lagomorphs, many species still remain unstudied, especially in the family Ochotonidae. In this study, to the best of our knowledge, the presence of Trypanosomatidae was demonstrated for the first time in Mongolian pikas and their fleas.

Flea-borne diseases are diverse and globally distributed. Among them, plague, bartonellosis, and typhus, which are caused by *Yersinia pestis*, bartonellae, *Rickettsia typhi*, and* R. felis*, are considered as especially severe human infections transmitted by fleas [14–16]. In the past, the *T. lewisi* clade was considered as a rat-specific (*R. norvegicus* and *R. rattus*) group of pathogens, transmitted by rat fleas (*Xenopsylla cheopis* and *Nosopsyllus fasciatus*) and non-pathogenic to humans [17, 18]. Recently, an increasing number of cases involving humans infected with members of the *T. lewisi* clade have been reported around the world, along with the emergence of a fatal infection [18–21]. In 2015, the resistance of this parasite to the lysis by normal human serum was reported [22]. In the present study, we detected a previously unknown *Trypanosoma* genotype from both Mongolian pikas and fleas, which shared 100% sequence identity. Our findings suggest that the flea *F. elatoides elatoides* parasitizing Mongolian pikas may serve as a carrier for this hemoflagellate. In addition, splenomegaly was noted in *Trypanosoma*-positive individuals, compared with the normal spleen of uninfected pikas (Additional file 3). These results necessitate further studies to evaluate the clinicopathological significance of *Trypanosoma* sp. pika.

Regarding vectors of Trypanosomatidae other than flies, *Blechomonas luni* was reported from the flea *C. globiceps*, *T. binneyi* from the leech, and *T. rhipicephalis* from the tick species *Rhipicephalus microplus* [23–25]. In general, members of the genus *Blechomonas* are associated with fleas as reservoirs or vectors [23]. Here a *B. luni*-like trypanosomatid parasite was detected in the flea species *F. elatoides elatoides* for the first time. On the basis of the topology of the *18S rRNA* gene phylogenetic tree and high bootstrap supports, it is likely that both *Trypanosoma* sp. pika and *B. luni*-like genotype represent hitherto unknown species.

To date, most studies on pathogens in pikas (especially in plateau pikas) focused on *Echinococcus shiquicus*, *Toxoplasma gondii*, H5N1 avian influenza virus, and H9N2 avian influenza virus [26–29]. Accordingly, Mongolian pikas may act as reservoirs for many other infectious agents besides trypanosomes. In light of this, it is very important to increase the number of target species in the family Ochotonidae that are indigenous to Asia, with collection of their ectoparasites, blood, and organ samples from both local animals and introduced wildlife, because these will likely continue to reveal the diversity and distribution of emerging pathogens impacting domestic animals, wildlife, and humans.

In conclusion, this study presents the first evidence of *Trypanosoma* sp. pika in pikas and fleas and of the *B. luni*-like genotype in fleas. Our findings expand the taxonomic diversity, geographical range, and host spectrum of Trypanosomatidae. Therefore, it is an important task for future studies to expand the scope of this research by exploring a wider spectrum of natural hosts and arthropod vectors of these blood parasites in Central Asia.

## Supplementary Information

**Additional file 1: **Map of northwestern China showing sampling sites and coordinates.

**Additional file 2: **Phylogenetic tree of Trypanosomatidae species from Mongolian pikas and their fleas, based on the *gGAPDH* gene. The new sequences provided in the present study are indicated by a black circle (followed by the accession number).

**Additional file 3: **Macroscopic appearance of splenomegaly in trypanosoma-infected Mongolian pika

## Data Availability

The sequences obtained and analyzed during the present study are deposited in the GenBank database under the accession numbers OR701822 (flea *18S rRNA* gene), OR690791, OR700018, and OR701933 (trypanosome *18S rRNA* gene), as well as PP199391-PP199393 (trypanosome *gGAPDH* gene).

## References

[1] Votýpka J, d’Avila-Levy CM, Grellier P, Maslov DA, Lukeš J, Yurchenko V. New approaches to systematics of trypanosomatidae: criteria for taxonomic (re)description. Trends Parasitol. 2015;31:460–9. 26433249 10.1016/j.pt.2015.06.015

[2] Frolov AO, Kostygov AY, Yurchenko V. Development of Monoxenous Trypanosomatids and Phytomonads in Insects. Trends Parasitol. 2021;37:538–51. 33714646 10.1016/j.pt.2021.02.004

[3] Koual R, Buysse M, Grillet J, Binetruy F, Ouass S, Sprong H, et al. Phylogenetic evidence for a clade of tick-associated trypanosomes. Parasit Vectors. 2023;16:3. 36604731 10.1186/s13071-022-05622-y PMC9817367

[4] Wang X, Liang D, Jin W, Tang M, Liu S, Zhang P. Out of Tibet: genomic perspectives on the evolutionary history of extant pikas. Mol Biol Evol. 2020;37:1577–92. 32027372 10.1093/molbev/msaa026

[5] Tang RX, Wang J, Li YF, Zhou CR, Meng GL, Li FJ, et al. Genomics and morphometrics reveal the adaptive evolution of pikas. Zool Res. 2022;43:813–26. 35993133 10.24272/j.issn.2095-8137.2022.072 PMC9486525

[6] Lambert JP, Zhang X, Shi K, Riordan P. The pikas of China: a review of current research priorities and challenges for conservation. Integr Zool. 2023;18:110–28. 34937133 10.1111/1749-4877.12615

[7] Ji N, Chen X, Liu G, Zhao S, Tan W, Liu G, et al. *Theileria*, *Hepatozoon* and *Taenia* infection in great gerbils (*Rhombomys opimus*) in northwestern China. Int J Parasitol Parasites Wildl. 2021;15:79–86. 33996439 10.1016/j.ijppaw.2021.04.002 PMC8099453

[8] Yin X, Zhao S, Yan B, Tian Y, Ba T, Zhang J, Wang Y. *Bartonella rochalimae*, *B. grahamii*, *B. elizabethae*, and *Wolbachia* spp. in fleas from wild rodents near the China-Kazakhstan border. Korean J Parasitol. 2019;57:553–9. 31715700 10.3347/kjp.2019.57.5.553 PMC6851259

[9] Mafie E, Saito-Ito A, Kasai M, Hatta M, Rivera PT, Ma XH, et al. Integrative taxonomic approach of trypanosomes in the blood of rodents and soricids in Asian countries, with the description of three new species. Parasitol Res. 2019;118:97–109. 30353232 10.1007/s00436-018-6120-3

[10] Austen JM, Van Kampen E, Egan SL, O'Dea MA, Jackson B, Ryan UM, et al. First report of *Trypanosoma dionisii* (Trypanosomatidae) identified in Australia. Parasitology. 2020;147:1801–1809. 32981530 10.1017/S0031182020001845 PMC10317716

[11] Zhao SS, Li HY, Yin XP, Liu ZQ, Chen CF, Wang YZ. First detection of *Candidatus Rickettsia barbariae* in the flea *Vermipsylla alakurt* from north-western China. Parasit Vectors. 2016;9:325. 27267467 10.1186/s13071-016-1614-2 PMC4895814

[12] Simo G, Njitchouang GR, Njiokou F, Cuny G, Asonganyi T. Genetic characterization of *Trypanosoma brucei* circulating in domestic animals of the Fontem sleeping sickness of Cameroon. Microbes Infect. 2012;14:651–8. 22387499 10.1016/j.micinf.2012.02.003

[13] Vourchakbé J, Tiofack ZAA, Kante TS, Mpoame M, Simo G. Molecular identification of *Trypanosoma brucei gambiense* in naturally infected pigs, dogs and small ruminants confirms domestic animals as potential reservoirs for sleeping sickness in Chad. Parasite. 2020;27:63. 33206595 10.1051/parasite/2020061 PMC7673351

[14] Krige AS, Thompson RCA, Wills A, Burston G, Thorn S, Clode PL. “A flying start”: wildlife trypanosomes in tissues of Australian tabanids (Diptera: Tabanidae). Infect Genet Evol. 2021;96:105152. 34823027 10.1016/j.meegid.2021.105152

[15] Bitam I, Dittmar K, Parola P, Whiting MF, Raoult D. Fleas and flea-borne diseases. Int J Infect Dis. 2010;14:e667–76. 20189862 10.1016/j.ijid.2009.11.011

[16] Eisen RJ, Gage KL. Transmission of flea-borne zoonotic agents. Annu Rev Entomol. 2012;57:61–82. 21888520 10.1146/annurev-ento-120710-100717

[17] Garcia HA, Rangel CJ, Ortíz PA, Calzadilla CO, Coronado RA, Silva AJ, et al. Zoonotic trypanosomes in rats and fleas of Venezuelan slums. EcoHealth. 2019;16:523–33. 31583491 10.1007/s10393-019-01440-4

[18] Ortiz PA, Garcia HA, Lima L, da Silva FM, Campaner M, Pereira CL, Jittapalapong S, Neves L, Desquesnes M, Camargo EP, Teixeira MMG. Diagnosis and genetic analysis of the worldwide distributed *Rattus*-borne *Trypanosoma* (*Herpetosoma*) *lewisi* and its allied species in blood and fleas of rodents. Infect Genet Evol. 2018;63:380–90. 28882517 10.1016/j.meegid.2017.09.001

[19] Sarataphan N, Vongpakorn M, Nuansrichay B, Autarkool N, Keowkarnkah T, Rodtian P, Stich RW, Jittapalapong S. Diagnosis of a *Trypanosoma lewisi*-like (*Herpetosoma*) infection in a sick infant from Thailand. J Med Microbiol. 2007;56:1118–21. 17644723 10.1099/jmm.0.47222-0 PMC3066167

[20] Verma A, Manchanda S, Kumar N, Sharma A, Goel M, Banerjee PS, et al. *Trypanosoma lewisi* or *T. lewisi*-like infection in a 37-day-old Indian infant. Am J Trop Med Hyg. 2011;85:221–4. 21813838 10.4269/ajtmh.2011.11-0002 PMC3144816

[21] Jain P, Goyal V, Agrawal R. An atypical *Trypanosoma lewisi* infection in a 22-day-old neonate from India: an emergent zoonosis. Indian J Pathol Microbiol. 2023;66:199–201. 36656242 10.4103/ijpm.ijpm_449_22

[22] Lun ZR, Wen YZ, Uzureau P, Lecordier L, Lai DH, Lan YG, et al. Resistance to normal human serum reveals *Trypanosoma lewisi* as an underestimated human pathogen. Mol Biochem Parasitol. 2015;199:58–61. 25858024 10.1016/j.molbiopara.2015.03.007

[23] Votýpka J, Suková E, Kraeva N, Ishemgulova A, Duží I, Lukeš J, Yurchenko V. Diversity of trypanosomatids (Kinetoplastea: Trypanosomatidae) parasitizing fleas (Insecta: Siphonaptera) and description of a new genus Blechomonas gen. n. Protist. 2013;164:763–81. 24113136 10.1016/j.protis.2013.08.002

[24] Paparini A, Macgregor J, Irwin PJ, Warren K, Ryan UM. Novel genotypes of *Trypanosoma binneyi* from wild platypuses (*Ornithorhynchus anatinus*) and identification of a leech as a potential vector. Exp Parasitol. 2014;145:42–50. 25045852 10.1016/j.exppara.2014.07.004

[25] Morel N, Thompson CS, Rossner MV, Mangold AJ, Nava S. A *Trypanosoma* species detected in *Rhipicephalus* (*Boophilus*) *microplus* ticks from Argentina. Ticks Tick Borne Dis. 2021;12:101573. 33007666 10.1016/j.ttbdis.2020.101573

[26] Fan YL, Lou ZZ, Li L, Yan HB, Liu QY, Zhan F, et al. Genetic diversity in *Echinococcus shiquicus* from the plateau pika (*Ochotona curzoniae*) in Darlag County, Qinghai, China. Infect Genet Evol. 2016;45:408–14. 27282470 10.1016/j.meegid.2016.06.016

[27] Zhang XX, Lou ZZ, Huang SY, Zhou DH, Jia WZ, Su C, Zhu XQ. Genetic characterization of *Toxoplasma gondii* from Qinghai vole, Plateau pika and Tibetan ground-tit on the Qinghai-Tibet Plateau China. Parasit Vectors. 2013;6:291. 24192458 10.1186/1756-3305-6-291 PMC3852027

[28] Zhou J, Sun W, Wang J, Guo J, Yin W, Wu N, et al. Characterization of the H5N1 highly pathogenic avian influenza virus derived from wild pikas in China. J Virol. 2009;83:8957–64. 19553321 10.1128/JVI.00793-09 PMC2738197

[29] Yu Z, Cheng K, Sun W, Xin Y, Cai J, Ma R, et al. Lowly pathogenic avian influenza (H9N2) infection in Plateau pika (*Ochotona curzoniae*), Qinghai Lake China. Vet Microbiol. 2014;173:132–5. 25069623 10.1016/j.vetmic.2014.07.002

