# Supplementary figures and images for: Novel trypanosomatid species detected in Mongolian pikas (Ochotona pallasi) and their fleas in northwestern China
Source: Parasit Vectors. 2024 Mar 22;17:152. doi: 10.1186/s13071-024-06216-6 (PMC10958963; doi:10.1186/s13071-024-06216-6)

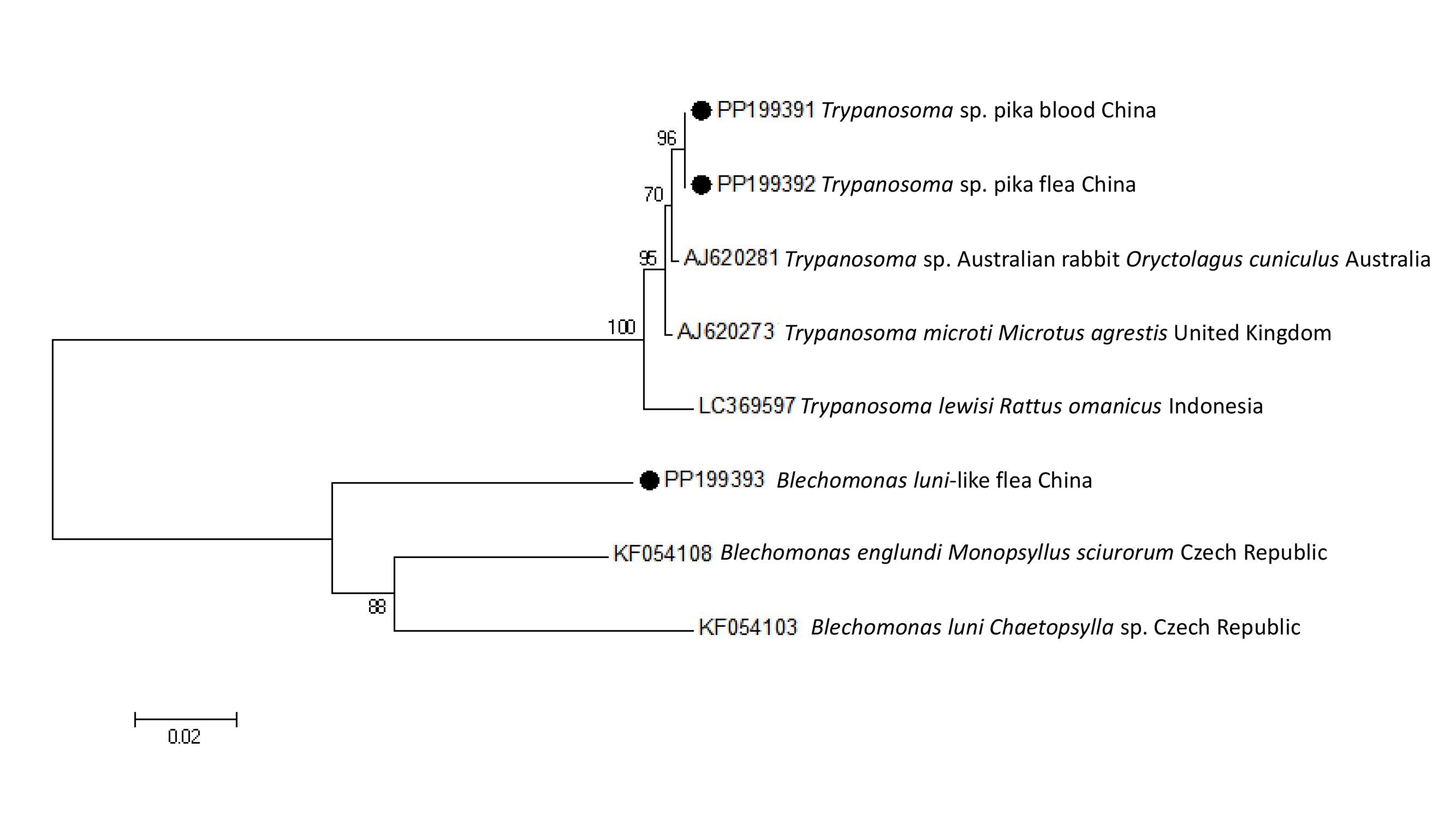

Supplement: Supplementary file 2 — Additional file 2: Phylogenetic tree of Trypanosomatidae species from Mongolian pikas and their fleas, based on the gGAPDH gene. The new sequences provided in the present study are indicated by a black circle (followed by the accession number). [file 13071_2024_6216_MOESM2_ESM.tif]

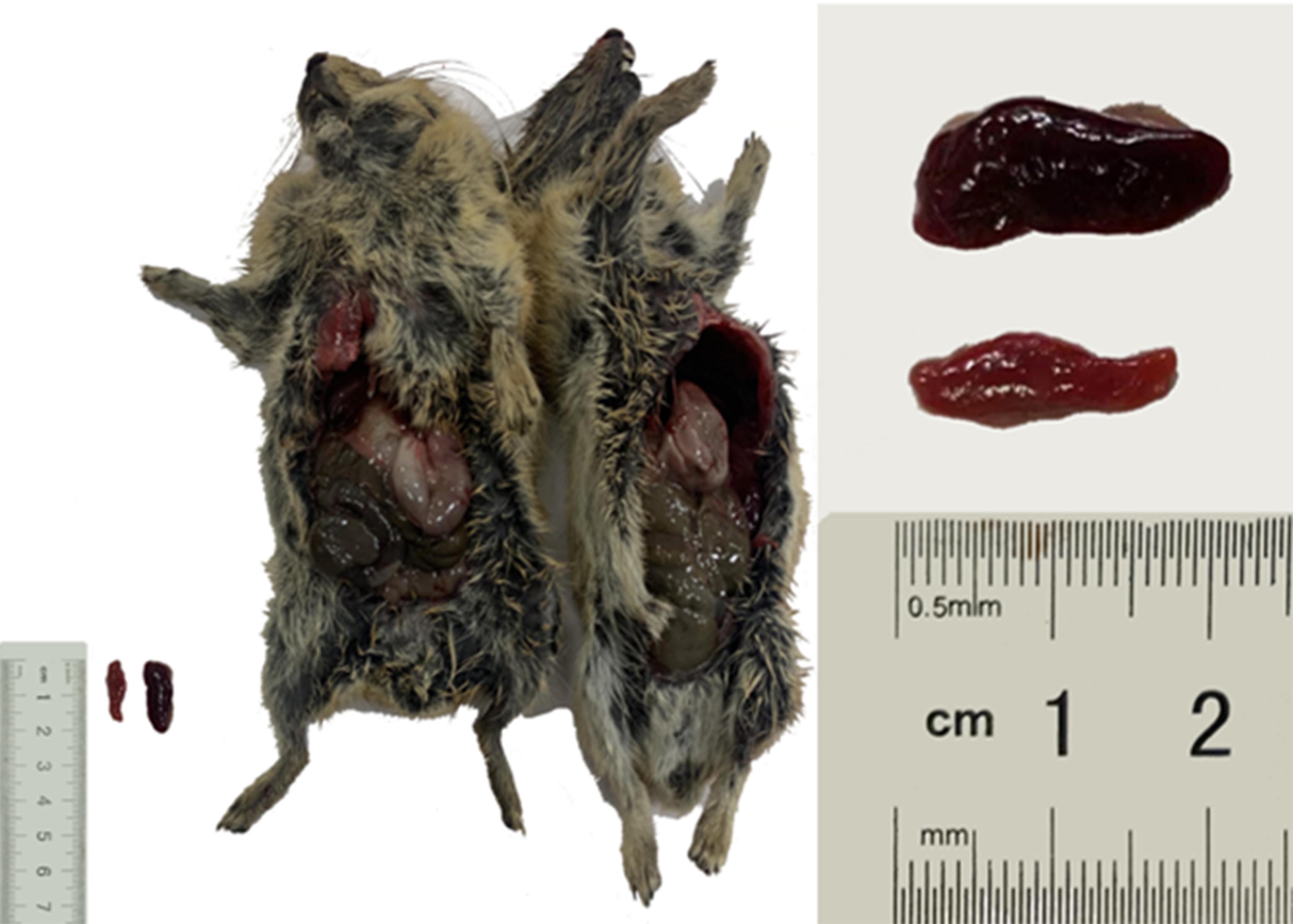

Supplement: Supplementary file 3 — Additional file 3: Macroscopic appearance of splenomegaly in trypanosoma-infected Mongolian pika [file 13071_2024_6216_MOESM3_ESM.tif]
